# How I do it: percutaneous transforaminal endoscopic discectomy for lumbar disk herniation

**DOI:** 10.1007/s00701-018-3723-5

**Published:** 2018-11-12

**Authors:** Paul R. A. M. Depauw, Pravesh S. Gadjradj, John S. Soria van Hoeve, Biswadjiet S. Harhangi

**Affiliations:** 1 Department of Neurosurgery, Elisabeth-TweeSteden Hospital, Tilburg, The Netherlands; 2 Department of Neurosurgery, Erasmus MC: University Medical Center Rotterdam, S-Gravendijkwal 230 NA-2110, 3015 CE Rotterdam, The Netherlands; 3 Department of Neurosurgery, Leiden University Medical Center, Leiden, The Netherlands

**Keywords:** Lumbar disk herniation, Discectomy, Transforaminal, Endoscopy

## Abstract

**Background:**

Percutaneous transforaminal endoscopic discectomy (PTED) has emerged as a less invasive technique to treat symptomatic lumbar disk herniation (LDH). PTED is performed under local anesthesia with the advantage of immediate intraoperative feedback of the patient. In this paper, the technique is described as conducted in our hospital.

**Methods:**

PTED is performed under local anesthesia in prone position on thoracopelvic supports. The procedure is explained stepwise: e.g. marking, incision, introduction of the 18-gauge needle and guidewire to the superior articular process, introduction of the TomShidi needle and foraminotomy up to 9 mm, with subsequently removal of disk material through the endoscope. Scar size is around 8 mm.

**Conclusion:**

PTED seems a promising alternative to conventional discectomy in patients with LDH and can be performed safely.

**Electronic supplementary material:**

The online version of this article (10.1007/s00701-018-3723-5) contains supplementary material, which is available to authorized users.

## Relevant surgical anatomy

Percutaneous transforaminal endoscopic discectomy (PTED) is performed through Kambin’ triangle of the neuroforamen [3]. The Kambin’s triangle is a three-dimensional anatomic right triangle over the dorsolateral intervertebral disk of the lumbar spine. In a two-dimensional plane from a lateral view, the boundaries of the Kambin’s triangle are the superior endplate of the inferior vertebral body (base of the triangle), the superior articulating facet (the height of the triangle), and the exiting superior nerve root (the hypotenuse of the triangle).

This landmark has been described many times in the context of fluoroscopic access to the epidural space. Access to this space has been utilized for minimally invasive steroid injections, which aim directly at the nerve root of interest. This posterolateral approach to the lumbar spine has been considered to be a safe area of access to the disk space.

The endoscopic view is from the anterolateral side of the dura and the descending nerve root in the spinal canal. The most important anatomical landmark for this procedure is the superior articular process (SAP) of the underlying vertebra. For the subarticular and foraminal lumbar disk herniation (LDH) the SAP is the anchoring point for the TomShidi needle after which the drilling is performed and the endoscope is introduced. The exiting nerve root is an important structure to avoid. During this procedure this nerve is however seldom seen since it is located in the upper part of the foramen.

## Description of the technique

**Fig. 1:**
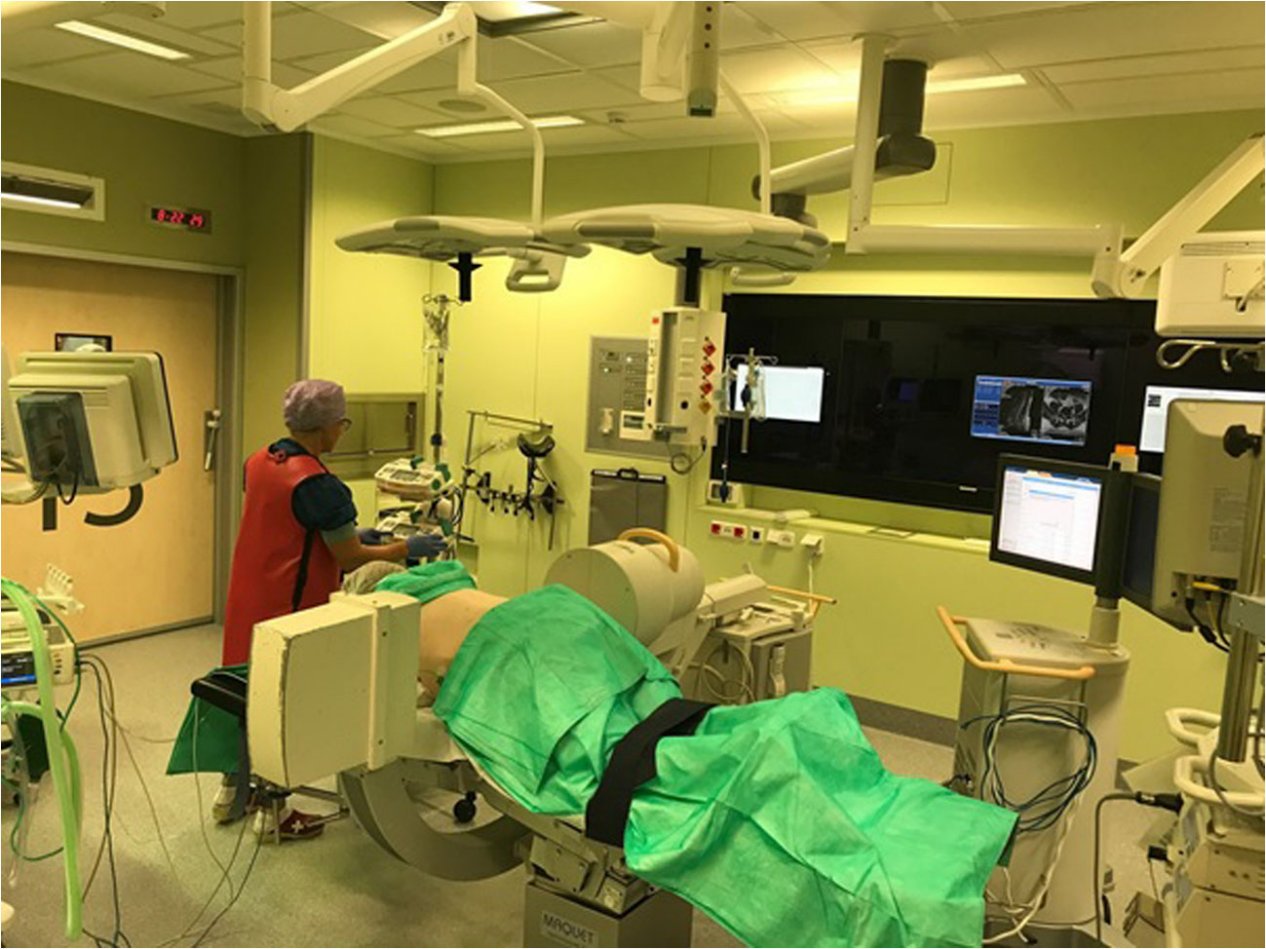
The operative setup during the PTED procedure. The patient is placed prone on a radiolucent table on thoracopelvic supports

The patient is positioned prone with support of chest and pelvis to free the abdomen (Fig. 1). True lateral and anteroposterior (AP) fluoroscopy is mandatory and should preferably be carried out with minimal manipulation of the C-arm. The SAP should be visualized clearly before draping. Both SAPs should project as one on the lateral view. The endplates of the adjacent vertebral body’s should also project as one. PTED is performed under conscious sedation so that patient can give feedback during surgery.

**Fig. 2:**
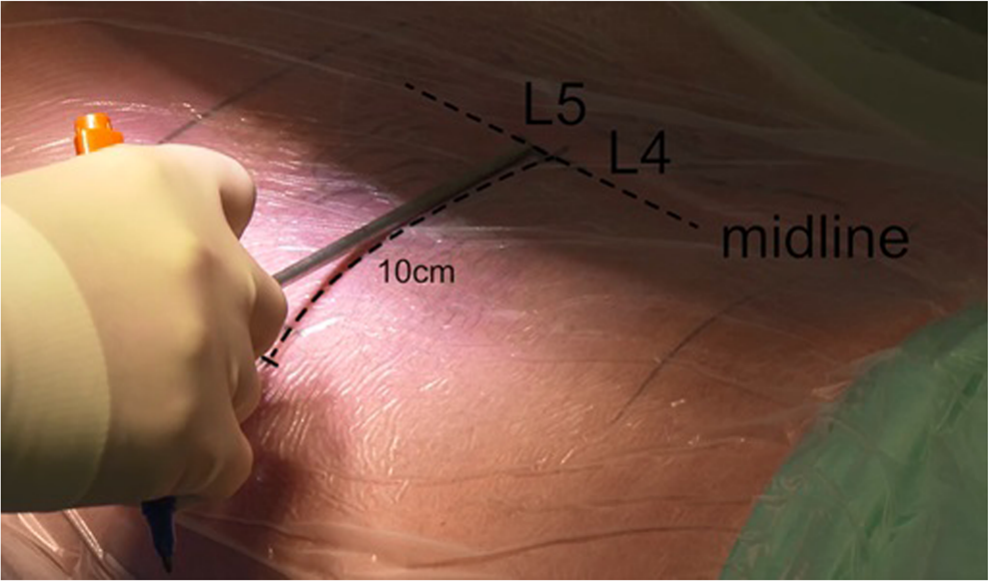
Marking of the trajectory. For level L4–L5, the incision is placed 10 cm from the midline

The SAP is approached using a long 18-gauge needle and with AP and lateral fluoroscopy. The angle should be approximately 40–50° in craniocaudal direction to the SAP of the lower level for L5–S1 and 30–40° and 20–25° for levels L4–5 and L3–4, respectively (Fig. 2). The skin incision is marked 8–13 cm from the midline depending on the level of surgery L4–L5 is marked 10 cm from the midline, while for L5–S1 incision is marked 12 cm from midline [7]. A preoperative planning of the trajectory on MRI is always needed to approach the herniated disk in a straight line. In case of a L5–S1 disk herniation a preoperative X-ray is preferred to evaluate the height of the iliac crest in reference to the disk space of L5-S1.

**Fig. 3:**
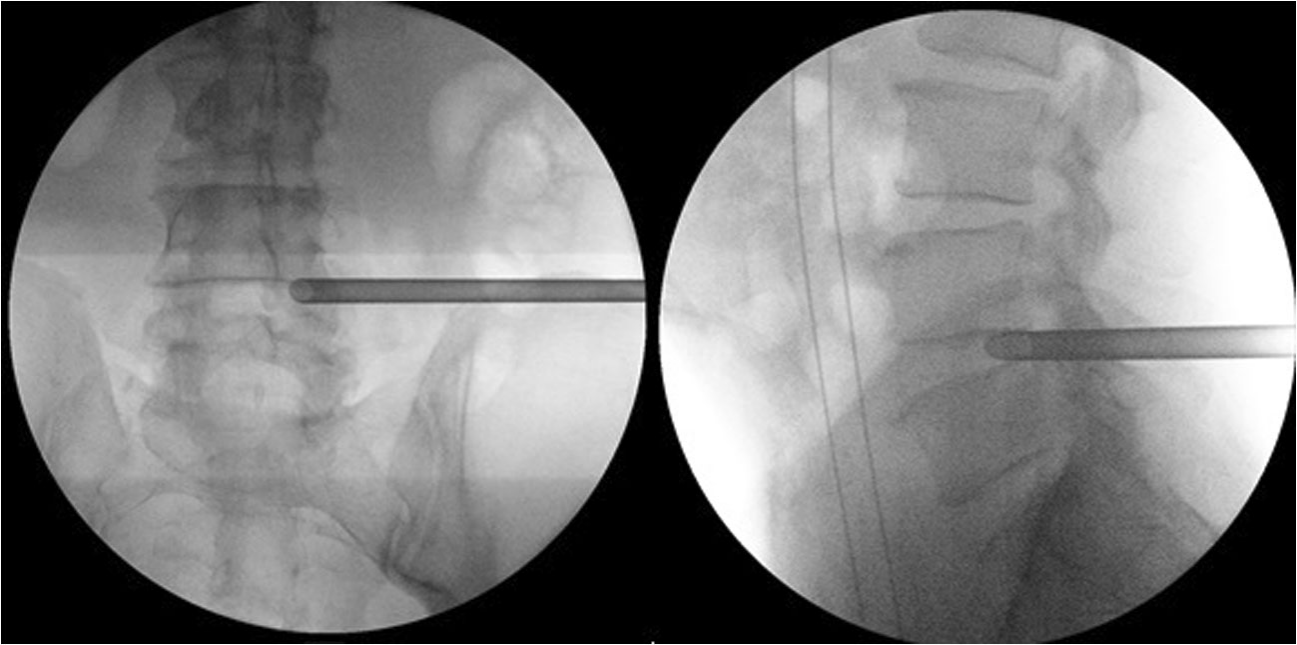
With AP and lateral fluoroscopy the to be operated lumbar disk is approached

**Fig. 4:**
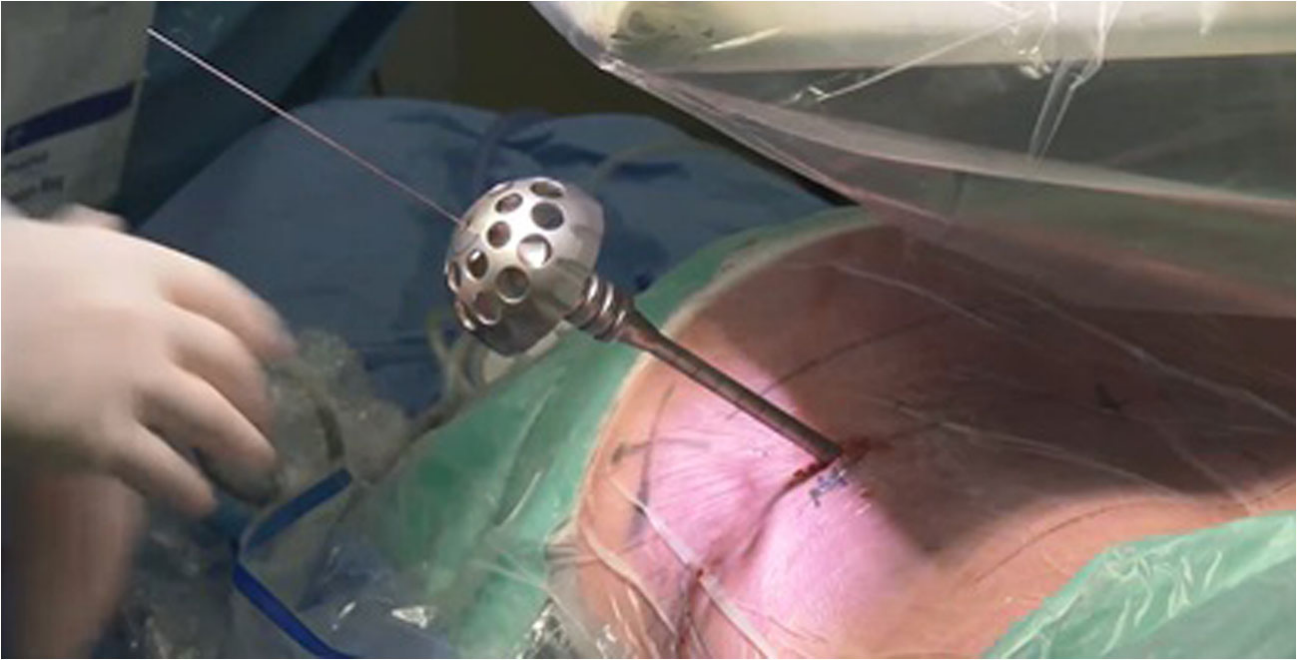
With hand drills over the guidewire, a foraminotomy up to 9 mm can be performed

The 18-G needle is introduced and the level and direction of the needle to the SAP is checked with true AP and lateral view images. When the right entry point is marked, the skin is infiltrated with local anesthetics and subsequently the trajectory is infiltrated. It is important to infiltrate to the thoracolumbar fascia since the introduction of the cannulated conical rods can be painful at this point. The SAP is reached by manipulation and evaluation of the tactile feedback of the surgeon and the position is checked by AP and lateral X-ray (Fig. 3). The facet joint is then anesthetized with lidocaine. The guidewire is introduced through the 18-G needle. The needle is removed, while the guidewire stays in place. The trajectory is widened with cannulated conical rods. The guidewire is removed, after introducing the cannulated TomShidi needle. With the sharp TomShidi needle, the top of the SAP is gently perforated in the direction of Kambin’ triangle while entering the epidural space with the tip. Next, the sharp needle is replaced by a blunt needle tip to fully enter the epidural space preventing damage to neuronal structures. Thereafter, a 1-mm guidewire is introduced to guide the drills (Fig. 4).

**Fig. 5:**
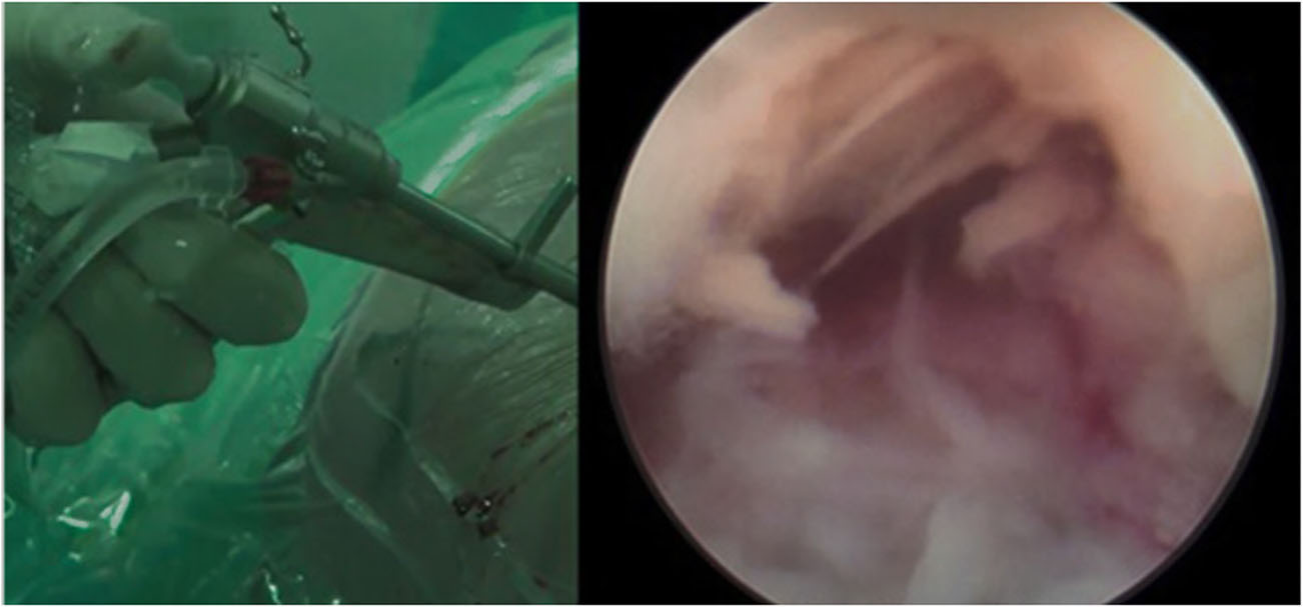
The endoscope is placed into the working channel, showing fragments of the herniated disk on the right

Drilling through the SAP in the direction of the disk is started with a 4-mm disposable drill. In this transarticular approach, the foramen is then widened up to 8 or 9 mm with different reusable drills with a blunt tip in order to prevent damage to neuronal structures. Drills are introduced anticlockwise to prevent muscle damage. After drilling, the working channel is introduced over the dilatator. The tip of the working channel is aimed at the posterior longitudinal ligament (PLL). The working channel is anchored in the drilled bony trajectory. The opening of the working channel is directed to the dura*.* Hereafter, the 30° angled endoscope is introduced and proper position of the camera is checked (Fig. 5). A pressure regulated pump is used for rinsing with 9% saline. In the superior part of the endoscopic view, the facet joint should be located and the PLL should be at the inferior part. Now, loose tissue and disk fragments are removed gently. The evaluation of the amount of decompression is debatable but concluded sufficient when there is a clear increase of pulsations of the dura or when there is a clear view of a pulsating nerve root. Venous bleeding can be stopped by increasing the pressure in the working channel or using bipolar coagulation. After removing the rinsing water by introducing the largest cannulated rod, the working channel is removed. The skin is closed intracutaneously.

## Indications

PTED is performed for minimally invasive treatment of LDH. A period of non-surgical treatment, however, should always forego surgical treatment [5]. In almost all cases, lumbar discectomy is an elective procedure. Reasons for emergency discectomy are progressive motor deficits or cauda equina syndrome.

## Limitations

In experienced hands, PTED can be used to treat all types of LDHs [1]. As already mentioned by Kambin in 1996, proper patient selection is mandatory for a successful outcome in endoscopic disk surgery. This also accounts for his approach. It is experienced that sequestered and migrated herniations (upward or downward) and large central herniations at L5–S1 in individuals with elevated iliac crest are technically harder to perform surgery on [4]. In case of concomitant lumbar spinal stenosis, except for foramen stenosis, the authors prefer to perform open microdiscectomy (OM).

## How to avoid complications

In contrast to (OM), PTED has a steep learning curve [9]. Good three-dimensional anatomical knowledge of the facet joint, ligaments, and nerves is mandatory. Currently, the PTED technique is under investigation in a large randomized controlled trial: PTED study [8]. In this trial, spine surgeons started training with a cadaveric workshop, followed by 20 procedures under strict supervision of an experienced surgeon (BSH). Furthermore, adequate sedation and feedback of the patient is mandatory to prevent nerve damage.

## Specific perioperative considerations

Positioning with thoracopelvic supports is essential to avoid spinal venous congestion. Furthermore, when patients experience much pain, make sure to use sufficient local anesthetic especially at the fascia thoracolumbalis and SAP. When removing disk material, the amount of removed material should match the amount on the preoperative MRI.

## Specific information to give the patient about surgery and potential risks

Patients should be counseled on specific features of PTED. It is important for patient to understand that they will undergo surgery under conscious sedation and that they can be safely discharged 2 hours after surgery. Furthermore, patients should understand that there is a lack of high-quality evidence on the equivalence of clinical outcomes of PTED compared to OM. However, to the best of our knowledge, PTED is expected to have similar clinical outcomes as compared to OM in the treatment of LDH [2, 6]. Potential complications of PTED are except conversion to OM similar as those of OM and may include dural tear, nerve root injury, deep or superficial wound infection, progressive or persistent neurological complaints, and recurrent LDH.

## Summary key points

1. Proper instruction of patients on the perioperative experience is crucial for adequate feedback during surgery.
2. Surgery is performed under conscious sedation with local infiltration of the operative trajectory.
3. Decompression is performed through a skin incision of 8 mm. The incision should be marked 10 and 12 cm from the midline for levels L4–L5 and L5–S1, respectively.
4. After introducing the 18-G needle, make sure, while anesthetizing the SAP, to not anesthetize the nerve root. This may lead to loss of adequate feedback during decompression.
5. The TomShidi needle is placed on top of the SAP. The needle should be directed in to Kabin’s triangle.
6. Drills are placed over the guidewire to dilatate the neuroforamen. The foraminotomy can be performed up to 9 mm.
7. After foraminotomy, a working cannula is placed. Through this cannula, the endoscope is introduced through which loose disk material can be removed with a rongeur.
8. Decompression can be considered sufficient when the nerve shows pulsations simultaneously with the heart rate and when the removed material matches the expected amount as based on MRI.
9. Patients can be discharged safely on the day of surgery.
10. A robust randomized controlled non-inferiority trial on the cost-effectiveness of PTED is underway.

## Electronic supplementary material

Video 1 The PTED procedure is shown stepwise in a patient with a symptomatic lumbar disk herniation on L4-L5. (MP4 227,861 kb)

## References

[1] Gadjradj PS, van Tulder MW, Dirven CM, Peul WC, Harhangi BS (2016). Clinical outcomes after percutaneous transforaminal endoscopic discectomy for lumbar disc herniation: a prospective case series. Neurosurg Focus.

[2] Gibson JNA, Subramanian AS, Scott CEH (2017). A randomised controlled trial of transforaminal endoscopic discectomy vs microdiscectomy. Eur Spine J.

[3] Kambin P, Sampson S (1986) Posterolateral percutaneous suction-excision of herniated lumbar intervertebral disks - report of interim results. Clin Orthop Relat Res 37–43 3720102

[4] Kambin P, Zhou LQ (1996). History and current status of percutaneous arthroscopic disc surgery. Spine.

[5] Peul WC, van Houwelingen HC, van den Hout WB, Brand R, Eekhof JA, Tans JT, Thomeer RT, Koes BW, Leiden-The Hague Spine Intervention Prognostic Study G (2007). Surgery versus prolonged conservative treatment for sciatica. N Engl J Med.

[6] Rasouli MR, Rahimi-Movaghar V, Shokraneh F, Moradi-Lakeh M, Chou R (2014) Minimally invasive discectomy versus microdiscectomy/open discectomy for symptomatic lumbar disc herniation. Cochrane Database Syst Rev 10.1002/14651858.CD010328.pub2 PMC10961733 25184502

[7] Schubert M, Hoogland T (2005). Endoscopic transforaminal nucleotomy with foraminoplasty for lumbar disk herniation. Oper Orthop Traumatol.

[8] Seiger A, Gadjradj PS, Harhangi BS, van Susante JL, Peul WC, van Tulder MW, de Boer MR, Rubinstein SM (2017). PTED study: design of a non-inferiority, randomised controlled trial to compare the effectiveness and cost-effectiveness of percutaneous transforaminal endoscopic discectomy (PTED) versus open microdiscectomy for patients with a symptomatic lumbar disc herniation. BMJ Open.

[9] Yasargil MG (1977). Microsurgical operation of herniated lumbar-disk. Acta Neurochir.

